# The story of the pandemic: navigating our way between optimism and pessimism

**DOI:** 10.1007/s11299-020-00232-6

**Published:** 2020-05-25

**Authors:** Rona Unrau

**Affiliations:** Max Planck Institute for Human Development, Berlin, Germany

Any narrative on the current pandemic is necessarily a fiction. Present-day dictators may be squeezing the numbers and hiding the facts, but, to crudely paraphrase George Orwell, as much as they disapprove of the pandemic, they cannot prevent its consequences. Contrary to many headlines and our understandable yearning for closure, not a soul on earth knows how the virus will develop and what the ultimate repercussions will be. Are the cheery slogans we cling to—“everything will be fine”—healthy optimism or merely wishful thinking from minds attuned to Hollywood happy ends? Are the dire apocalyptic warnings something we will look back to as prophetic visions or as yet another instance of mindless panic?

Our world may indeed end up a (slightly) better place. Less mobility may lead to less global warming. The air is said to be much cleaner in many a city across the world.^1^ Less disposable wealth may give people pause to reconsider the dynamics of unhemmed capitalism and consumerism. At least in surveys people have indicated their intentions to reduce their indiscriminate buying and simplify their lifestyles.^2^ Increased awareness of our own mortality may lead to more compassion and responsibility for each other. Although the problem has often been reduced to a conflict between old and weak versus strong and young, it is astonishing how many young people devote time to helping those classified as “risk groups” and who willingly comply with the rules of a lockdown, in spite of the potentially high personal costs.^3^ The stronger flow of information and increased transparency of political and scientific discourse may lead to stronger democratic participation and interest in acquiring empirically sound knowledge. The “nerdy” discussions in many a mainstream newspaper forum on reproduction values and other statistical niceties certainly attest to a new appreciation of mathematics in everyday life.

Or the world may end up a (decidely) worse place. Less mobility may lead to increased nationalism and blinkered views of the world. We already have enough shameful examples of this in both the US and Europe alone.^4^ Increased awareness of our mortality may lead to less compassion and more egoistic efforts to survive at the cost of others. While worrying about where to buy their next package of toilet paper or how to survive two months without a haircut, have middle-class Westerns kept other problems on their radar, such as the millions reported to be in danger of starving in India from the lockdown or African countries having just fractions of the number of ICU beds available in industrial countries?^5^ The stronger flow of information and transparency of political and scientific discourse may ignite the flickering flames of populism and weaken faith in democracy. Although the noise is no doubt larger than the actual numbers of dissenters, loud and sometimes violent protests are on the rise.

Or perhaps the world will end up yet another muddy mix of both the good and the bad, as humans make use of their indispensable double-edged sword of adaptive resilience: reverting as quickly as possible to the “normal” while accepting some of the changes to their lives as givens. It is the paradox of our incoherently coherent existence to date.

Over the past weeks I have marvelled at the unprecedented admission of uncertainty by both politicians and scientists. If my memory serves me well, never has there been such an outburst of collective brainstorming in public. Each and every idea thrown into the arena echoes a yes–but that cannot be resolved. Even the “surefire” statistics that have entered lay vocabulary, such as exponential growth, R_0_ values, or other markers, are under debate in the shifting sea of uncertainty of deciding how to deal with the virus. This is science and politics in their most honest and transparent form. For those of us used to equations of authority with absolute knowledge, whose pre-pandemic readings more likely included *Immorality Inc.* and *Homo deus* than the *Ars moriendi*, this apparent helplessness may be psychologically devastating.

At the same time, these yes–but issues reveal how marvellously intricate the fabric of our civilization is, tightly woven to encompass our psychologies and cultures, our ideologies and ideals, our shadows and beams of sunshine. But they also reveal why it is so extremely difficult to remove destructive threads deep within that fabric, and why it may well be foolhardy to rigorously tear out entire strips of old cloth, as ugly as they may be. As economic and health crises threaten to tear apart our civilization, we might therefore do better to act like observant gardeners in a historical park, carefully pruning, grafting, weeding, fertilizing, and planting anew in synch with changing climatic conditions.

This mixed bag of metaphors is necessarily vague, with no guarantee that the tapestry will shine or the garden flourish. They too are quite possibly mere fictions. What can be offered are some words of the late Vaclav Havel, former dissident and last president of Czechoslovakia, written in an entirely different context:

Hope is definitely not the same thing as optimism. It is not the conviction that something will turn out well, but the certainty that something makes sense, regardless of how it turns out. […]

It is also this hope, above all, which gives the strength to live and continually to try new things, even in conditions that seem hopeless as ours do, here and now.^6^

This hope, to me, is that which has been shared and nurtured over the ages by the best of science, politics, art, and religion—and by numerous individuals outside these categories. In the context of the pandemic, it is a hope that most or at least many of us will avoid the pitfalls of denial and complacency, anger and aggression, self-interest and extreme consumerism and, together, instead continually try new things to shape the future for the better, no matter what transpires.

## Footnotes

1 Bauwens, Compernolle, Stavrakou et al. (8 May 2020) Impact of coronavirus outbreak on NO_2_ pollution assessed using TROPOMI and OMI observations. *Geophysical Research Letters*. 10.1029/2020GL087978.

2 E.g., https://www.mckinsey.de/~/media/McKinsey/Locations/Europe%20and%20Middle%20East/Deutschland/News/Presse/2020/2020-05-07%20Consumer%20Sentiment%20Wave%205/200505_Consumer_Sentiment_Survey_Wave5_Germany.ashx.

3 E.g., https://www.un.org/africarenewal/web-features/coronavirus/meet-10-young-people-leading-covid-19-response-their-communities.

4 https://www.piie.com/blogs/trade-and-investment-policy-watch/covid-19-trumps-curbs-exports-medical-gear-put-americans-and; https://www.reuters.com/article/health-coronavirus-germany-exports/germany-bans-export-of-medical-protection-gear-due-to-coronavirus-idUSL8N2AX3D9.

5 India: https://www.fes-asia.org/news/stories-of-hunger-indias-lockdown-is-hitting-the-poorest/; Africa: https://www.rfi.fr/en/africa/20200403-lack-of-covid-19-treatment-and-critical-care-could-be-catastrophic-for-africa.

6 Václav Havel in Disturbing the Peace: A conversation with Karel Hvížďala, (Knopf, 1990), p. 181. Originally published 1986. Translated from the Czech by Paul Wilson.

